# Chinese SLE Treatment and Research group (CSTAR) registry: Clinical significance of thrombocytopenia in Chinese patients with systemic lupus erythematosus

**DOI:** 10.1371/journal.pone.0225516

**Published:** 2019-11-20

**Authors:** N. Jiang, M. Li, M. Zhang, J. Xu, L. Jiang, L. Gong, F. Wu, J. Gu, J. Zhao, Y. Xiang, Z. Wang, Y. Zhao, X. Zeng, CSTAR co-authors

**Affiliations:** 1 Department of Rheumatology, Peking Union Medical College Hospital, Peking Union Medical College & Chinese Academy of Medical Sciences, Key Laboratory of Rheumatology and Clinical Immunology, Ministry of Education, Beijing, China; 2 Department of Rheumatology, Jiangsu Provincial People's Hospital, Nanjing, Jiangsu, China; 3 Department of Rheumatology, the First Affiliated Hospital of Anhui Medical University, Hefei, Anhui, China; 4 Department of Rheumatology, Zhongshan Hospital Affiliated to Fudan University, Shanghai, China; 5 Department of Rheumatology, the General Hospital of Tianjin Medical University, Tianjin, China; 6 Department of Rheumatology, Capital Institute of Pediatrics, Beijing, China; 7 Department of Rheumatology, the Third Affiliated Hospital of Sun Yat-sen University, Guangzhou, Guangdong, China; Peking University First Hospital, CHINA

## Abstract

**Objectives:**

To investigate the prevalence, clinical characteristics, and prognosis of thrombocytopenia (TP) in Chinese patients with systemic lupus erythematosus (SLE).

**Methods:**

The study was conducted based on the Chinese SLE Treatment and Research group (CSTAR) registry. Thrombocytopenia was defined as the platelet count<100,000/mm^3^ at enrollment. Severe thrombocytopenia was defined as the platelet count<50,000/mm^3^. The prevalence of SLE-related TP, the associations of thrombocytopenia with demographic data, organ involvements, laboratory findings, disease activity, damage, and mortality were investigated.

**Results:**

Of 2104 patients with SLE, 342 patients (16.3%) were diagnosed with thrombocytopenia. The prevalence of neuropsychiatric SLE, vasculitis, myositis, nephritis, mucocutaneous lesions, pleuritis, fever, leukocytopenia and hypocomplementemia were significantly higher in patients with thrombocytopenia (p<0.05). SLE disease activity index (SLEDAI) was significantly higher in patients with thrombocytopenia (p<0.05). Multivariate analysis showed that leukocytopenia (OR = 2.644), lupus nephritis (OR = 1.539), hypocomplementemia (OR = 1.497) and elevated SLEDAI (OR = 1.318) were independently associated with thrombocytopenia (p<0.05). Long disease duration (OR = 1.006) was an independent risk factor of severe thrombocytopenia, while anti-rRNP (OR = 0.208) was an independent protective factor of severe thrombocytopenia (p<0.05). Long disease duration was an independent risk factor of mortality in patients with thrombocytopenia (RR = 1.006). The 6-year survival of patients with thrombocytopenia was significantly lower than patients without thrombocytopenia (88.2% vs. 95.5%).

**Conclusions:**

Thrombocytopenia was a common manifestation of SLE and was associated with leukocytopenia, nephritis and severe disease activity. Severe thrombocytopenia tended to occur in long-term and relatively inactive SLE. Patients with SLE-related thrombocytopenia has a decreased long-term survival rate. Long disease duration was an independent risk factor of mortality in patients with thrombocytopenia.

## Introduction

Systemic lupus erythematosus (SLE) is a complicated autoimmune disease which can affect almost all systems and organs. Skin, muscle skeletal system, hematological system and kidney are most frequently involved systems in Chinese patients [1]. Thrombocytopenia (TP) is a common hematological disorder in patients with SLE. The prevalence is estimated to range from 10% to 40% according to published literatures [2–5].

The association between thrombocytopenia and characteristics of SLE patients has been investigated in several studies. Thrombocytopenia has been shown to be associated with other severe clinical manifestations and poor prognosis in patients with SLE [6–8]. However, multicenter data regarding thrombocytopenia in Chinese SLE patients are limited.

Chinese SLE Treatment and Research group (CSTAR), which is supported by Chinese National Key Technology Research and Development Program, developed the first on-line registry of Chinese patients with SLE. This registry has described major clinical characteristics and related manifestations in Chinese patients, such as pulmonary arterial hypertension and serositis [9–10]. It also provides the opportunity to investigate the epidemiological and clinical features of patients with SLE-related thrombocytopenia.

## Methods

### Patient recruitment

Based on the CSTAR online registry, the study was conducted at 104 high-rank rheumatology centers in 30 provinces across China. As the leading center, Peking Union Medical College Hospital (PUMCH) takes substantial responsibilities for the training, communication and funding for the registry. This study was approved by the Medical Ethics Committee of Peking Union Medical College Hospital (PUMCH), which was the leading research center. Most centers accepted Ethics Committees (EC) from PUMCH as the leading site, some approved by their own EC, included Beijing Tongren Hospital, the General Hospital of Tianjing Medical University, and the Second Affiliated Hospital of Guangzhou Medical College. Written informed consents were provided by all patients before their enrollments into the CSTAR registry. Between April 2009 and February 2010, the CSTAR registry recruited 2104 Chinese SLE patients who fulfilled the 1997 SLE classification criteria revised by the ACR [11].

### Definition of thrombocytopenia

**Fig 1:**
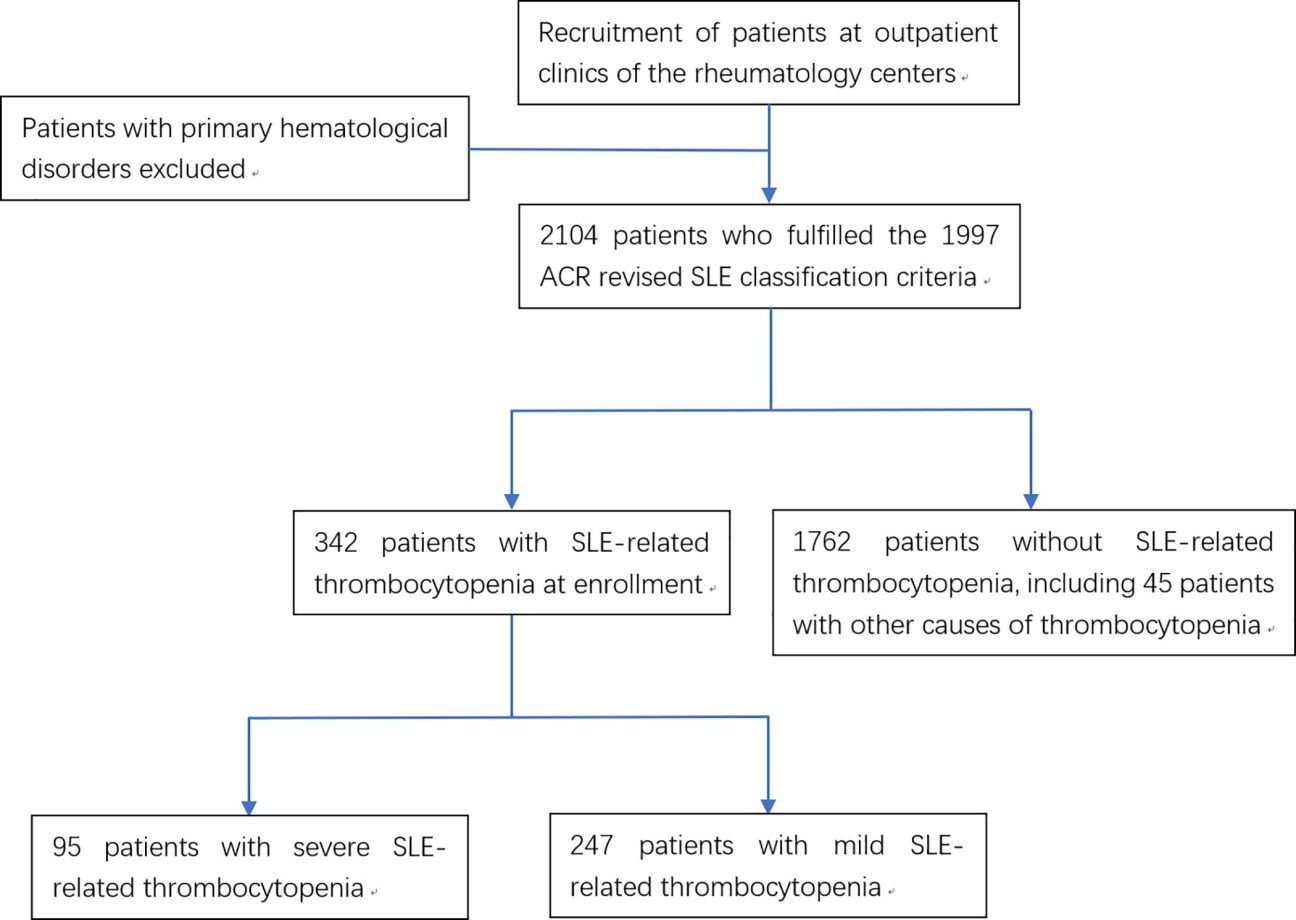
Recruitment and exclusion flowchart.

Lupus-related thrombocytopenia was defined as the platelet count less than 100,000/mm^3^ at baseline. The diagnosis was made only if other causes, such as primary hematological disorders, viral infection and drug-induced thrombocytopenia, were excluded by the physicians at enrollment. Patients with thrombocytopenia were further categorized as having mild or severe thrombocytopenia. The same definition of severe thrombocytopenia as the LUMINA cohort [6] (platelet count less than 50,000/mm^3^) was used in this study. The recruitment and exclusion processes were shown in Fig 1.

### Data collection

All CSTAR centers used the same protocol-directed methods to provide uniform evaluations and to record the patients’ data. Investigators received training on diagnosis confirmation, disease activity evaluation, data input and data quality control. In this 6-year longitudinal study, demographic, clinical and laboratory data were collected. Systemic involvements, including neuropsychiatric SLE, vasculitis, arthritis, myositis, lupus nephritis, mucocutaneous lesions, pleuritic and fever, were defined according to the SLE Disease Activity Index (SLEDAI) [12]. Laboratory data included white blood cell counts, complement levels and autoantibodies. Antinuclear antibodies (ANA), anti-double-stranded (ds)DNA, anti-Smith (Sm), anti-SSA/Ro, anti-SSB/La, anti-ribonucleoprotein (RNP), anti-ribosomal (anti-r) RNP and anti-phospholipid antibodies were measured in all patients at local laboratories. SLE disease activity was evaluated in all patients by SLEDAI. Damage was measured with the Systemic Lupus International Collaborating Clinics/American College of Rheumatology Damage Index (SDI) [13] at the last visit. Mortality was recorded within six years. Data were collected between April 2009 and March 2016. The authors did not have access to information that could identify individual participants during or after data collection.

### Statistical analysis

A case-control approach was used to compare parameters between patients with and without thrombocytopenia, and between patients with severe thrombocytopenia and mild thrombocytopenia (platelet count less than 100,000/mm^3^ but great than or equal to 50,000/mm^3^). All analyses were conducted using the SPSS 19.0 statistical package (SPSS, Chicago, USA). Variables were described using counts and/or percentages or medians and ranges. Student’s t test was used to compare quantitative data. Chi-squared test was used for the comparison of categorical data between the two groups. Variables with P values <0.05 in the univariate analyses were further investigated using multivariate binary logistic regression analysis. Mortality was compared between patients with and without thrombocytopenia, and between patients with severe thrombocytopenia and mild thrombocytopenia, respectively, using Kaplan-Meier survival analysis and multivariate Cox regression. P values <0.05 were considered to be statistically significant.

## Results

### Patients and demographics

From April 2009 to February 2010, 2104 Chinese patients with SLE who fulfilled four or more of the 1997 ACR revised SLE classification criteria were registered into the CSTAR cohort. Of these patients, 342 (16.3%) had thrombocytopenia at baseline. In patients with thrombocytopenia, mean (SD) platelet count was 78.8 (65.1) ×10^9^/mm^3^, mean (SD) age at onset was 30.5 (12.1) years, age at diagnosis was 32.0 (12.4) years. The mean (SD) disease duration was 41.5 (64.6) months. As shown in Table 1, there was no significant difference in age, gender or disease duration between patients with thrombocytopenia and those without.

**Table 1:** Demographic and clinical features of patients with or without thrombocytopenia.

	*Thrombocytopenia*		*P value*
	*Yes (n = 342)*	*No (n = 1762)*	
Platelet count (1,000/mm^3^)	78.8±65.1	206.1±76.5	
Sex, female	311 (90.9%)	1603 (91.0%)	0.981
Age at onset (years)	30.5±12.1	29.4±12.3	0.150
Age at diagnosis (years)	32.0±12.4	30.5±12.5	0.056
Disease duration (months)	41.5±64.6	42.1±57.7	0.874
NPSLE	29 (8.5%)	89 (5.1%)	**0.012**
Vasculitis	36 (10.5%)	107 (6.1%)	**0.003**
Arthritis	95 (27.8%)	537 (30.5%)	0.319
Myositis	13 (3.8%)	35 (2.0%)	**0.040**
Lupus nephritis	195 (57.0%)	654 (37.1%)	**<0.001**
Mucocutaneous involvement	195 (57.0%)	893 (50.7%)	**0.032**
Pleuritis	48 (14.0%)	149 (8.5%)	**<0.001**
Fever	99 (28.9%)	352 (20.0%)	**<0.001**

NPSLE: neuropsychiatric systemic lupus erythematosus.

### Clinical manifestations

The clinical manifestations are shown in Table 1. In univariate analysis, the prevalence of neurological involvement, vasculitis, myositis, nephritis, mucocutaneous involvement, pleuritic and fever were significantly higher in patients with thrombocytopenia than those without (P<0.05).

### Laboratory findings

As shown in Table 2, the prevalence of leukocytopenia and hypocomplementemia were significantly higher in patients with thrombocytopenia in univariate analysis.

**Table 2:** Laboratory findings of patients with or without thrombocytopenia.

	*Thrombocytopenia*		*P value*
	*Yes (n = 342)*	*No (n = 1762)*	
Leukocytopenia	152 (44.4%)	352 (20.0%)	**<0.001**
Hypocomplementemia	280 (81.9%)	1119 (63.5%)	**<0.001**
ANA	334 (97.7%)	1730 (98.2%)	0.517
Anti-dsDNA	95 (27.8%)	519 (29.5%)	0.532
Anti-Sm	65 (19.0%)	284 (16.1%)	0.189
Anti-RNP	29 (8.5%)	160 (9.1%)	0.722
Anti-SSA/Ro	93 (27.2%)	407 (23.1%)	0.104
Anti-SSB/La	40 (11.7%)	184 (10.4%)	0.492
Anti-rRNP	41/165 (24.8%)	212/847 (25.0%)	0.959
APL	88/175 (50.3%)	326/760 (42.9%)	0.076

ANA: anti-nuclear antibodies; anti-dsDNA: anti-double-stranded DNA; anti-RNP: anti-ribonucleoprotein; anti-rRNP: anti-ribosomal RNP; anti-Sm: anti-Smith; APL: anti-phospholipid.

### SLE disease activity

SLEDAI was significantly higher in thrombocytopenia group than in non-thrombocytopenia group (13.2±7.5 vs. 9.0±6.7, p<0.001).

### Multivariate analysis

Multivariate analysis revealed that leukocytopenia, lupus nephritis, hypocomplementemia and elevated SLEDAI were independent related factors of thrombocytopenia in patients with SLE (all p<0.05), as shown in Table 3.

**Table 3:** Multivariable analysis on related factors of thrombocytopenia in SLE.

	*Odds ratio (95% CI)*	*P value*
NPSLE	1.155 (0.697–1.914)	0.577
Vasculitis	1.157 (0.730–1.832)	0.535
Myositis	1.281 (0.637–2.575)	0.487
Lupus nephritis	1.539 (1.121–2.111)	**0.008**
Mucocutaneous involvement	0.831 (0.633–1.092)	0.185
Pleuritis	1.041 (0.709–1.527)	0.838
Fever	1.034 (0.775–1.380)	0.821
Leukocytopenia	2.644 (2.035–3.435)	**<0.001**
Hypocomplementemia	1.497 (1.077–2.082)	**0.016**
SLEDAI	1.318 (1.069–1.624)	**0.010**

NPSLE: neuropsychiatric systemic lupus erythematosus; SLEDAI: Systemic Lupus Erythematosus Disease Activity Index.

### Prognosis

**Fig 2:**
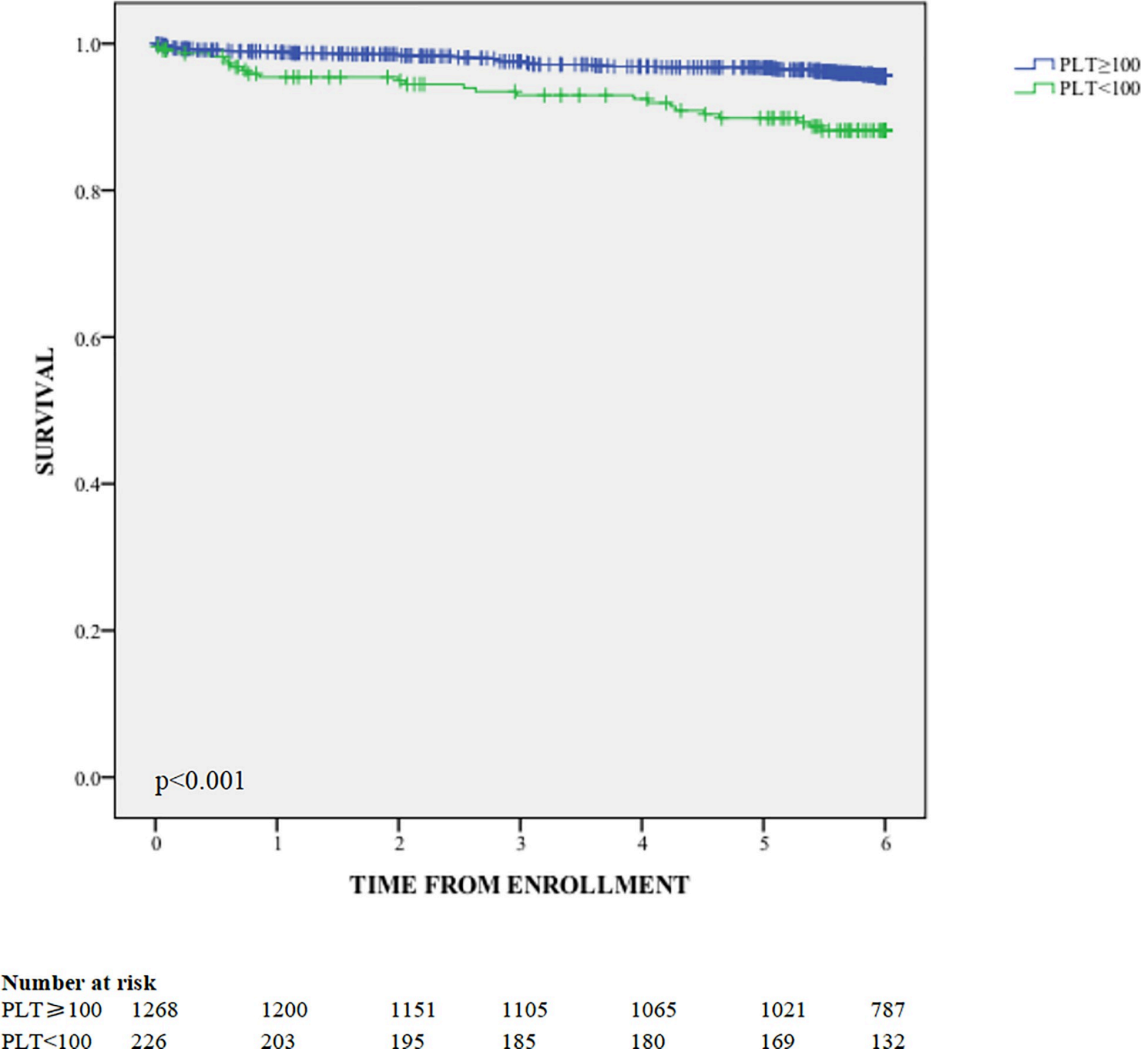
Survival of patients with and without thrombocytopenia.

Of 2104 patients, 1494 had long-term follow-up data. No difference in damage at last visit was observed between thrombocytopenia (n = 226) and non-thrombocytopenia (n = 1268) group (0.36±0.66 vs. 0.30±0.60, p = 0.168). The 6-year survival of patients with thrombocytopenia was significantly lower than patients without thrombocytopenia (88.2% vs. 95.5%, p<0.001, as shown in Fig 2). In multivariate Cox regression (Table 4), thrombocytopenia, sex, age of onset, disease duration, baseline damage and factors related with TP (nephritis, leukocytopenia, hypocomplementemia and SLEDAI) were analyzed. Male sex, older age of onset, and nephritis at baseline were revealed to be risk factors of mortality.

**Table 4:** Multivariate Cox regression on risk factors of mortality in patients with SLE.

	*P value*	*RR*	*95% CI of RR*	
			*Lower limit*	*Upper limit*
Sex (male)	**0.003**	3.491	1.521	8.013
Age of onset	**0.001**	1.042	1.017	1.069
Disease duration	0.476	1.027	.954	1.106
Lupus nephritis	**0.019**	3.096	1.204	7.962
Leukocytopenia	0.224	1.683	0.728	3.891
Hypocomplementemia	0.285	0.622	0.260	1.485
SLEDAI	0.741	0.989	0.924	1.058
Baseline damage	0.139	1.872	0.815	4.301
Thrombocytopenia	0.517	1.346	0.548	3.306

RR: relative risk; CI: confidence interval; SLEDAI: Systemic Lupus Erythematosus Disease Activity Index.

### Comparison of patients with severe and mild thrombocytopenia

As shown in Table 5, 95 patients (27.8%) had severe thrombocytopenia. Of these patients, 87 (91.6%) were female. Their mean (SD) platelet count was 23.4 (14.2) ×10^9^/mm^3^, mean (SD) age at onset was 31.9 (12.2) years, age at diagnosis was 33.5(12.2) years. Their mean (SD) disease duration was 60.9 (80.8) months, which was obviously longer than patients with mild thrombocytopenia. There was no significant difference in age or gender between patients with severe or mild thrombocytopenia. The prevalence of nephritis and mucocutaneous involvement were significantly lower in patients with severe thrombocytopenia than patients with mild thrombocytopenia (P<0.05).

**Table 5:** Demographic and clinical features of patients with mild or severe thrombocytopenia.

	*Thrombocytopenia*		*P value*
	*Severe (n = 95)*	*Mild (n = 247)*	
Platelet count (1,000/mm^3^)	23.4±14.2	74.6±14.1	
Sex, female	87 (91.6%)	224 (90.7%)	0.797
Age at onset (years)	31.9±12.2	30.0±12.2	0.219
Age at diagnosis (years)	33.5±12.2	31.5±12.5	0.172
Disease duration (months)	60.9±80.8	34.0±55.6	**0.005**
NPSLE	8 (8.4%)	21 (8.5%)	0.981
Vasculitis	9 (9.5%)	27 (10.9%)	0.694
Arthritis	24 (25.3%)	71 (28.7%)	0.520
Myositis	1 (1.1%)	12 (4.9%)	0.183
Lupus nephritis	46 (48.4%)	149 (60.3%)	**0.046**
Mucocutaneous involvement	44 (46.3%)	151 (61.1%)	**0.013**
Pleuritis	11 (11.6%)	37 (15.0%)	0.417
Fever	21 (22.1%)	78 (31.6%)	0.084

NPSLE: neuropsychiatric systemic lupus erythematosus.

As shown in Table 6, the prevalence of leukocytopenia, hypocomplementemia, positive anti-dsDNA and anti-rRNP antibodies were significantly lower in patients with severe thrombocytopenia in univariate analysis (P<0.05).

**Table 6:** Laboratory findings of patients with mild or severe thrombocytopenia.

	*Thrombocytopenia*		*P value*
	*Severe (n = 95)*	*Mild (n = 247)*	
Leukocytopenia	30 (31.6%)	122 (49.4%)	**0.003**
Hypocomplementemia	69 (72.6%)	211 (85.4%)	**0.006**
ANA	91 (95.8%)	243 (98.4%)	0.307
Anti-dsDNA	12 (12.6%)	83 (33.6%)	**<0.001**
Anti-Sm	13 (13.7%)	52 (21.1%)	0.120
Anti-RNP	8 (8.4%)	21 (8.5%)	0.981
Anti-SSA/Ro	25 (26.3%)	68 (27.5%)	0.821
Anti-SSB/La	12 (12.6%)	28 (11.3%)	0.738
Anti-rRNP	3/39 (7.7%)	38/128 (29.7%)	**0.005**
APL	26/50(52.0%)	62/125 (49.6%)	0.774

ANA: anti-nuclear antibodies; anti-dsDNA: anti-double-stranded DNA; anti-RNP: anti-ribonucleoprotein; anti-rRNP: anti-ribosomal RNP; anti-Sm: anti-Smith; APL: anti-phospholipid.

SLEDAI was significantly lower in severe thrombocytopenia group than in mild thrombocytopenia group (11.2±7.5 vs. 14.0±7.4, p = 0.002). Baseline SDI was significantly lower in severe thrombocytopenia group than in mild thrombocytopenia group (0.08±0.32 vs. 14.0±7.4, p = 0.002).

As shown in Table 7, further multivariate analysis revealed that long disease duration was an independent risk factor of severe thrombocytopenia. Anti-rRNP was an independent protective factor of severe thrombocytopenia in patients with SLE.

**Table 7:** Multivariable analysis on related factors of severe thrombocytopenia in patients with systemic lupus erythematosus.

	*Odds ratio (95% CI)*	*P value*
Disease duration (months)	1.006 (1.000–1.012)	**0.041**
Lupus nephritis	0.590 (0.202–1.703)	0.335
Mucocutaneous involvement	0.967 (0.368–2.539)	0.945
Leukocytopenia	0.923 (0.352–2.419)	0.871
Hypocomplementemia	0.561 (0.194–1.626)	0.287
Anti-dsDNA	0.348 (0.092–1.316)	0.120
Anti-rRNP	0.208 (0.054–0.802)	**0.023**
SLEDAI	0.950 (0.855–1.056)	0.343

anti-dsDNA: anti-double-stranded DNA; anti-RNP: anti-ribonucleoprotein; anti-rRNP: anti-ribosomal RNP.

**Fig 3:**
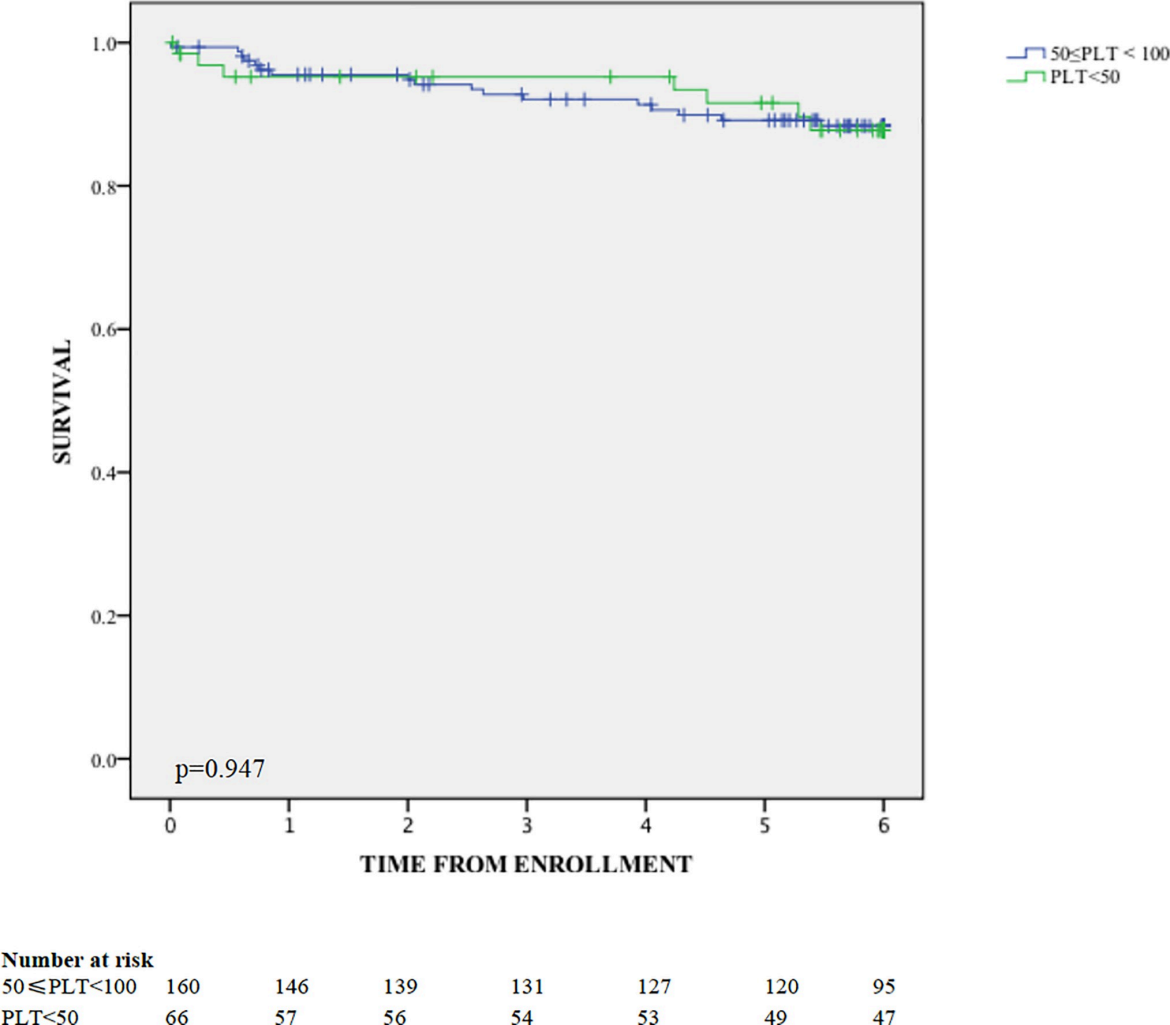
Survival of patients with mild and severe thrombocytopenia.

No difference in SDI at last visit was observed between patients with severe thrombocytopenia (n = 66) and mild thrombocytopenia (n = 160) (0.21±0.57 vs. 0.34±0.60, p = 0.141). The 6-year survival showed no difference between patients with severe and mild thrombocytopenia (88.3% vs. 87.8%, p = 0.947, as shown in Fig 3). In multivariate Cox regression (Table 8), severe or mild thrombocytopenia, sex, age of onset, disease duration, baseline damage and factors related with TP (nephritis, leukocytopenia, hypocomplementemia and SLEDAI) were analyzed. Only long disease duration was revealed to be a risk factor of mortality.

**Table 8:** Multivariate Cox regression on risk factors of mortality in patients with SLE-related TP.

	*P value*	*RR*	*95% CI of RR*	
			*Lower limit*	*Upper limit*
Sex (male)	0.294	2.017	0.543	7.487
Age of onset	0.170	1.026	0.989	1.064
Disease duration	**0.025**	1.006	1.001	1.011
Lupus nephritis	0.531	0.734	0.279	1.931
Leukocytopenia	0.338	0.632	0.247	1.615
Hypocomplementemia	0.128	0.483	0.189	1.233
SLEDAI	0.539	1.023	0.952	1.099
Baseline damage	0.330	1.636	0.608	4.407
Thrombocytopenia	0.484	0.702	0.260	1.894

RR: relative risk; CI: confidence interval; SLEDAI: Systemic Lupus Erythematosus Disease Activity Index.

## Discussion

This study is a longitudinal observational study on the prevalence and clinical characteristics of SLE-related thrombocytopenia in Chinese patients with SLE. The results showed that leukocytopenia, lupus nephritis, hypocomplementemia and elevated SLEDAI were independently associated with thrombocytopenia. The 6-year survival of patients with thrombocytopenia was significantly lower than those without. Long disease duration was an independent risk factor of severe thrombocytopenia, while anti-rRNP was an independent protective factor of severe thrombocytopenia.

Of 2104 patients with SLE, 342 patients (16.3%) were diagnosed with thrombocytopenia. The prevalence is consistent with data from a Latin American cohort [5] and a US cohort [6]. Patients with thrombocytopenia tended to be younger in previous reports [6,14], but in our study there is no difference in age, gender or disease duration between patients with and without thrombocytopenia.

In clinical practice, we found that patients with thrombocytopenia often have active disease and other organ involvements. Results of the study approved this clinical experience. The SLE disease activity was assessed with SLEDAI, which was significantly higher in thrombocytopenia group in both univariable and multivariable analyses, indicating that patients with thrombocytopenia had more active disease. Univariable analysis revealed patients with thrombocytopenia tended to have more serious disease manifestations, including neuropsychiatric SLE, vasculitis, myositis, nephritis, mucocutaneous involvements, pleuritis, fever and leukocytopenia. Lupus nephritis and leukocytopenia were shown to be independently risk factors of thrombocytopenia in multivariable analysis. As we know, hypocomplementemia indicates active SLE, and is associated with lupus nephritis. Therefore, it is stand to reason that hypocomplementemia and lupus nephritis were revealed as independent risk factors of thrombocytopenia in this study. Thrombocytopenia is often accompanied by other hematologic disorders in clinical practice. In a Latin American cohort, SLE-related thrombocytopenia was found to be associated with previous thrombocytopenia and autoimmune hemolytic anemia [5]. In our study we found leukocytopenia was a strong predictor of thrombocytopenia in patients with SLE. The result is in accordance with clinical experience.

The association between APL and thrombocytopenia has been reported in the literatures [5–6,15–16]. In this study, the association between APL and thrombocytopenia was not proved, but some tendency was observed (p = 0.076). As we know, thrombocytopenia secondary to SLE occurs in several clinical situations, including immunologic thrombocytopenic purpura (ITP), antiphospholipid syndrome (APS), macrophage activation syndrome (MAS), thrombotic thrombocytopenic purpura (TTP), etc. Theoretically, some of the conditions are not related to APL. We infer this is the reason that the incidences of APL showed no disparity in the thrombocytopenia and non-thrombocytopenia groups in our study. Further analysis of the characteristics of populations with different causes of SLE–related thrombocytopenia may provide more details. The associations between thrombocytopenia and other autoantibodies, such as anti-dsDNA, anti-Sm, anti-SSA and anti-RNP antibodies, have been variably reported in the literatures [5–6,14,17]. These associations were not observed in our data. The differences were probably due to distinct ethnic groups and varied disease stages in different cohorts.

As for prognosis, Kaplan-Meire survival analysis showed that patients with thrombocytopenia had a significantly lower long-term survival, indicating patients with SLE-related thrombocytopenia has a worse prognosis. In multivariate Cox regression analysis, thrombocytopenia was not found to be an independent prognostic factor. Since some of the known confounders, such as poverty and cumulative steroid dose, were not included in our study, the conclusion is to be clarified in further studies. At the last visit, thrombocytopenia was not associated with damage accrual in our cohort. Similar results were observed in the LUMINA cohort [6] and the GLADEL cohort [5]. It was a limitation that bleeding events were not recorded in our study. By adding these data in future follow-ups in CSTAR cohort, we would have more information for understanding the relationship between thrombocytopenia and the prognosis of SLE.

When comparing the different characteristics of severe and mild thrombocytopenia, we found that severe thrombocytopenia tended to occur in patients with longer disease duration. In univariate analysis, lupus nephritis, mucocutaneous involvement, leukocytopenia, hypocomplementemia, anti-dsDNA antibody, anti-ribosome RNP antibody and elevated SLEDAI appeared to be protective for the patients against severe thrombocytopenia. Moreover, none of the clinical or laboratory factors showed higher incidence in patients with severe thrombocytopenia. Since hypocomplementemia, positive anti-dsDNA antibody and elevated SLEDAI indicate active SLE, we can infer that severe thrombocytopenia was inclined to occur in patients with relatively low disease activity. Fernández et al found disease activity measured by the SLAM-R score was associated with thrombocytopenia, but not with severe thrombocytopenia (platelet count<50,000/mm^3^) [6], which were very consistent with the results in our study. It is widely accepted that anti-rRNP is related to neuropsychiatric involvement. Thus, the protective effect of lupus nephritis, mucocutaneous involvement, leukocytopenia and positive anti-rRNP observed in this study indicated that severe thrombocytopenia tended to occur in patients with few other organ involvements. In multivariable logistic analysis, the protective relationship was observed in anti-rRNP, suggesting that patients with negative anti-rRNP should be intensively monitored of developing severe thrombocytopenia. More observations are needed to confirm the relationship between severe thrombocytopenia and anti-rRNP or organ involvements. When analyzing the prognosis, both mortality and damage at last visit were not different in patients with mild or severe thrombocytopenia, indicating the possible similar disease outcome in the two populations. In multivariate Cox regression in patients with SLE-related thrombocytopenia, severe thrombocytopenia was not an independent risk factor of mortality comparing to mild thrombocytopenia. Long disease duration was revealed to be a risk factor of mortality, indicating that patients with thrombocytopenia and long term SLE need to be carefully monitored. In LUMINA cohort, severe thrombocytopenia (compared with all the SLE patients with platelet count≥50,000/mm^3^, not with patients with mild thrombocytopenia) was found to be independently associated with damage accrual at the last visit, which was not observed in our study (data not shown). More observations are needed in future studies to confirm whether severe thrombocytopenia is associated with damage accrual.

The study has some limitations. First, the definition of thrombocytopenia in our study was based on short-term results of platelet count at baseline. Characteristics of patients with refractory thrombocytopenia was not investigated. Second, we did not have data regarding autoimmune hemolytic anemia (AIHA) and actual etiology of thrombocytopenia (i.e., antiphospholipid syndrome, thrombotic thrombocytopenic purpura, immune thrombocytopenic purpura) in this study, since they were not included in the CSTAR cohort. Third, antiplatelet antibodies were not tested in this study, since the test were not available in some centers.

In conclusion, this study is a report of SLE-related thrombocytopenia with so far the largest sample size. We described the prevalence and clinical features of thrombocytopenia in patients from 30 provinces across China. Data indicated that thrombocytopenia was a common manifestation of SLE, and was associated with leukocytopenia, lupus nephritis and high disease activity. Severe thrombocytopenia tended to occur in long-term and relatively quiet SLE, which has few other systemic involvement and low disease activity. Long disease duration was an independent risk factor of mortality in patients with thrombocytopenia. Patients with SLE-related thrombocytopenia has a decreased survival rate.

## Supporting information

S1 File STROBE_checklist. (DOCX)

S2 File Minimal Anonymized Data. (XLSX)
