# Supplementary material for: Chinese SLE Treatment and Research group (CSTAR) registry: Clinical significance of thrombocytopenia in Chinese patients with systemic lupus erythematosus
Source: PLoS One. 2019 Nov 20;14(11):e0225516. doi: 10.1371/journal.pone.0225516 (PMC6867648; doi:10.1371/journal.pone.0225516)
Supplement: S1 File — (DOCX) [file pone.0225516.s001.docx]

STROBE Statement—checklist of items that should be included in reports of observational studies

|  | Item No. | Recommendation | Page  No. | Relevant text from manuscript |
| --- | --- | --- | --- | --- |
| **Title and abstract** | 1 | (*a*) Indicate the study’s design with a commonly used term in the title or the abstract | 3 | A cross-sectional study was conducted based on the Chinese SLE Treatment and Research group (CSTAR) registry. |
|  |  | (*b*) Provide in the abstract an informative and balanced summary of what was done and what was found | 3 | The prevalence of SLE-related TP and the associations of thrombocytopenia with demographic data, organ involvements, laboratory findings and disease activity were investigated.  Multivariate analysis showed that leukocytopenia (OR=2.644), lupus nephritis (OR=1.539), hypocomplementemia (OR=1.497) and elevated SLEDAI (OR=1.318) were independently associated with thrombocytopenia (p<0.05). Long disease duration (OR=1.006) was an independent risk factor of severe thrombocytopenia, while anti-rRNP (OR=0.208) was an independent protective factor of severe thrombocytopenia (p<0.05). |
| Introduction | | | |  |
| Background/rationale | 2 | Explain the scientific background and rationale for the investigation being reported | 5 | Thrombocytopenia (TP) is a common hematological disorder in patients with SLE. The prevalence is estimated to range from 10% to 40% according to published literatures.  The association between thrombocytopenia and characteristics of SLE patients has been investigated in several studies. Thrombocytopenia has been shown to be associated with other severe clinical manifestations and poor prognosis in patients with SLE. |
| Objectives | 3 | State specific objectives, including any prespecified hypotheses | 5 | It also provides the opportunity to investigate the epidemiological and clinical features of patients with SLE-related thrombocytopenia. |
| Methods | | | |  |
| Study design | 4 | Present key elements of study design early in the paper | 7 | A case-control approach was used to compare parameters between patients with and without thrombocytopenia, and patients with and without severe thrombocytopenia. |
| Setting | 5 | Describe the setting, locations, and relevant dates, including periods of recruitment, exposure, follow-up, and data collection | 6 | Between April 2009 and February 2010, the CSTAR registry recruited 2104 Chinese SLE patients who fulfilled the 1997 SLE classification criteria revised by the ACR. |
| Participants | 6 | (*a*) *Cohort study*—Give the eligibility criteria, and the sources and methods of selection of participants. Describe methods of follow-up  *Case-control study*—Give the eligibility criteria, and the sources and methods of case ascertainment and control selection. Give the rationale for the choice of cases and controls  *Cross-sectional study*—Give the eligibility criteria, and the sources and methods of selection of participants | 6 | Lupus-related thrombocytopenia was defined as the platelet count less than 100,000/mm^3^ at baseline. Patients with thrombocytopenia were further categorized as having mild or severe thrombocytopenia. Severe thrombocytopenia was defined as the platelet count less than 50,000/mm^3^. |
|  |  | (*b*) *Cohort study*—For matched studies, give matching criteria and number of exposed and unexposed  *Case-control study*—For matched studies, give matching criteria and the number of controls per case | NA | NA |
| Variables | 7 | Clearly define all outcomes, exposures, predictors, potential confounders, and effect modifiers. Give diagnostic criteria, if applicable | NA | NA |
| Data sources/ measurement | 8* | For each variable of interest, give sources of data and details of methods of assessment (measurement). Describe comparability of assessment methods if there is more than one group | 7 | In this cross-sectional study, demographic, clinical and laboratory data were collected. Systemic involvements, including neuropsychiatric SLE, vasculitis, arthritis, myositis, lupus nephritis, mucocutaneous lesions, pleuritic and fever, were defined according to the SLE Disease Activity Index (SLEDAI). Laboratory data included white blood cell counts, complement levels and autoantibodies. Antinuclear antibodies (ANA), anti-double-stranded (ds)DNA, anti-Smith (Sm), anti-SSA/Ro, anti-SSB/La, anti-ribonucleoprotein (RNP), anti-ribosomal (anti-r) RNP and anti-phospholipid antibodies were measured in all patients at local laboratories. SLE disease activity was evaluated in all patients by SLEDAI. |
| Bias | 9 | Describe any efforts to address potential sources of bias | NA | NA |
| Study size | 10 | Explain how the study size was arrived at | NA | NA |

Continued on next page

| Quantitative variables | 11 | Explain how quantitative variables were handled in the analyses. If applicable, describe which groupings were chosen and why | NA | NA |
| --- | --- | --- | --- | --- |
| Statistical methods | 12 | (*a*) Describe all statistical methods, including those used to control for confounding | 7 | Student’s t test was used to compare quantitative data. Chi-squared test was used for the comparison of categorical data between the two groups. |
|  |  | (*b*) Describe any methods used to examine subgroups and interactions | NA | NA |
|  |  | (*c*) Explain how missing data were addressed | NA | NA |
|  |  | (*d*) *Cohort study*—If applicable, explain how loss to follow-up was addressed  *Case-control study*—If applicable, explain how matching of cases and controls was addressed  *Cross-sectional study*—If applicable, describe analytical methods taking account of sampling strategy | NA | NA |
|  |  | (*e*) Describe any sensitivity analyses | NA | NA |
| Results | | | | |
| Participants | 13* | (a) Report numbers of individuals at each stage of study—eg numbers potentially eligible, examined for eligibility, confirmed eligible, included in the study, completing follow-up, and analysed | 8 | 2104 Chinese patients with SLE who fulfilled four or more of the 1997 ACR revised SLE classification criteria were registered into the CSTAR cohort. Of these patients, 342 (16.3%) had thrombocytopenia at baseline. |
|  |  | (b) Give reasons for non-participation at each stage | NA | NA |
|  |  | (c) Consider use of a flow diagram | NA | NA |
| Descriptive data | 14* | (a) Give characteristics of study participants (eg demographic, clinical, social) and information on exposures and potential confounders | 8-9, 11-12 | Table 1 and 4 |
|  |  | (b) Indicate number of participants with missing data for each variable of interest | NA | NA |
|  |  | (c) *Cohort study*—Summarise follow-up time (eg, average and total amount) | NA | NA |
| Outcome data | 15* | *Cohort study*—Report numbers of outcome events or summary measures over time | NA | NA |
|  |  | *Case-control study—*Report numbers in each exposure category, or summary measures of exposure | 8-13 | Table 1, 2, 4, and 5 |
|  |  | *Cross-sectional study—*Report numbers of outcome events or summary measures | NA | NA |
| Main results | 16 | (*a*) Give unadjusted estimates and, if applicable, confounder-adjusted estimates and their precision (eg, 95% confidence interval). Make clear which confounders were adjusted for and why they were included | NA | NA |
|  |  | (*b*) Report category boundaries when continuous variables were categorized | NA | NA |
|  |  | (*c*) If relevant, consider translating estimates of relative risk into absolute risk for a meaningful time period | NA | NA |

Continued on next page

| Other analyses | 17 | Report other analyses done—eg analyses of subgroups and interactions, and sensitivity analyses | NA | NA |
| --- | --- | --- | --- | --- |
| Discussion | | | | |
| Key results | 18 | Summarise key results with reference to study objectives | 14 | This study is a prospective observational study on the prevalence and clinical characteristics of SLE-related thrombocytopenia in Chinese patients with SLE. The results showed that leukocytopenia, lupus nephritis, hypocomplementemia and elevated SLEDAI were independently associated with thrombocytopenia. Long disease duration was an independent risk factor of severe thrombocytopenia, while anti-rRNP was an independent protective factor of severe thrombocytopenia. |
| Limitations | 19 | Discuss limitations of the study, taking into account sources of potential bias or imprecision. Discuss both direction and magnitude of any potential bias | 15, 16, 17 | Further analysis of the characteristics of populations with different causes of SLE–related thrombocytopenia may provide more details.  More observations are needed to confirm the relationship between severe thrombocytopenia and anti-rRNP or organ involvements.  The association between thrombocytopenia and SLE outcome was not determined in this cross-sectional study. Follow-ups of CSTAR cohort may give the answer and provide more information for our understanding of the disease. |
| Interpretation | 20 | Give a cautious overall interpretation of results considering objectives, limitations, multiplicity of analyses, results from similar studies, and other relevant evidence | 16-17 | Data indicated that thrombocytopenia was a common manifestation of SLE, and was associated with leukocytopenia, lupus nephritis and severe disease activity. Severe thrombocytopenia tended to occur in long-term and relatively quiet SLE, which has few other systemic involvement and low disease activity. |
| Generalisability | 21 | Discuss the generalisability (external validity) of the study results | 16-17 | this study is a report of SLE-related thrombocytopenia with so far the largest sample size. We described the prevalenceand clinical features of thrombocytopenia in patients from 30 provinces across China. |
| Other information | |  | | |
| Funding | 22 | Give the source of funding and the role of the funders for the present study and, if applicable, for the original study on which the present article is based | 17 | This study was supported by the Chinese National Key Technology R&D Program, Ministry of Science and Technology (2017YFC0907601, 2017YFC0907602, 2017YFC0907603, 2008BAI59B02), the Chinese National High Technology Research and Development Program, Ministry of Science and Technology (2012AA02A513). |

*Give information separately for cases and controls in case-control studies and, if applicable, for exposed and unexposed groups in cohort and cross-sectional studies.

**Note:** An Explanation and Elaboration article discusses each checklist item and gives methodological background and published examples of transparent reporting. The STROBE checklist is best used in conjunction with this article (freely available on the Web sites of PLoS Medicine at http://www.plosmedicine.org/, Annals of Internal Medicine at http://www.annals.org/, and Epidemiology at http://www.epidem.com/). Information on the STROBE Initiative is available at www.strobe-statement.org.
